# Effectiveness of nighttime vs full-time bracing in the treatment of moderate-grade adolescent idiopathic scoliosis: a secondary analysis of the CONTRAIS trial

**DOI:** 10.2340/17453674.2025.43706

**Published:** 2025-06-04

**Authors:** Anastasios CHARALAMPIDIS, Elias DIARBAKERLI, Kourosh JALALPOUR, Acke OHLIN, Anna Aspberg AHL, Hans MÖLLER, Allan ABBOTT, Paul GERDHEM, for the CONTRAIS study group

**Affiliations:** 1 Department of Clinical Science, Intervention and Technology (CLINTEC), Karolinska Institutet, Stockholm; 2 Department of Reconstructive Orthopaedics, Karolinska University Hospital, Stockholm; 3 Clinical and Molecular Osteoporosis Unit, Department of Clinical Sciences, Malmö, Lund University, Lund; 4 Department of Orthopaedics, Ryhov hospital, Jönköping; 5 Stockholm Center for Spine Surgery, Stockholm; 6 Department of Health, Medicine and Caring Sciences, Unit of Physiotherapy, Linköping University, Linköping; 7 Department of Orthopaedics, Linköping University Hospital, Linköping; 8 Department of Orthopaedics and Hand Surgery, Uppsala University Hospital, Uppsala; 9 Department of Surgical Sciences, Uppsala University, Uppsala, Sweden

## Abstract

**Background and purpose:**

Data on effectiveness of nighttime bracing compared with full-time bracing in adolescent idiopathic scoliosis is scarce. We aimed to investigate risk of curve progression and surgery with nighttime bracing vs full-time bracing for patients with moderate-grade adolescent idiopathic scoliosis.

**Methods:**

Skeletally immature individuals with idiopathic scoliosis (25°–40°) treated with a nighttime brace as part of a parallel-group randomized controlled trial (RCT) were compared with non-participants treated with a full-time brace. In the case of curve progression of more than 6° in the nighttime brace group individuals were offered transition to a full-time brace. Surgery was offered if curve sizes were 45° or larger.

**Results:**

Median age at treatment start was 12.8 years (nighttime brace n = 45, full-time brace n = 44). Female sex (odds ratio [OR] 6.5, 95% confidence interval [CI] 1.1–37.4), lower Risser grade (OR 1.6, CI 1.01–2.7), and larger curve size at the beginning of brace treatment (OR 1.4, CI 1.2–1.5) increased the risk of curve progression to ≥ 45°. Major curves in the groups were similar at median 33 months’ follow-up (P = 0.7). After 94 months of follow-up, 11 patients in the nighttime brace group and 6 in the full-time brace group had undergone surgery (OR 2.0, CI 0.7–6.1).

**Conclusion:**

Nighttime bracing, including a possibility to transition to full-time brace in the case of progression, demonstrated comparable effectiveness in preventing curve progression, but a tendency to a higher risk of surgical treatment.

Brace treatment is the principal nonoperative treatment in patients with adolescent idiopathic scoliosis. Studies have shown the effectiveness of thoracolumbosacral orthosis (TLSO), worn for 18 to 22 hours daily, to prevent curve progression and need for fusion surgery [1,2]. Poor compliance and the psychological impact of brace wearing should be a cause for concern [3,4].

In this context, nighttime hyper-corrective bracing has been suggested as an alternative brace treatment in patients with adolescent idiopathic scoliosis. Recently, the results of our randomized trial CONservative TReatment for Adolescent Idiopathic Scoliosis (CONTRAIS) showed higher effectiveness of nighttime bracing, by preventing curve progression in up to 76% of cases, as compared with a control group who received physical activity only [5,6].

Nevertheless, studies investigating the effectiveness of nighttime bracing compared with full-time bracing are scarce and conflicting findings have been reported [7-13].

We aimed to compare treatment effectiveness between nighttime bracing and full-time bracing for patients with moderate-grade adolescent idiopathic scoliosis. We hypothesized that nighttime bracing was as effective as a full-time bracing in preventing curve progression and surgery in patients with moderate-grade adolescent idiopathic scoliosis.

## Methods

### Study design

The study cohort consists of individuals randomized to treatment with a hyper-corrective nighttime brace (custom-designed hypercorrective Boston scoliosis night brace [Camp Scandinavia, Helsingborg, Sweden]) vs nonparticipants, i.e., patients who did not consent to the randomized allocation and were treated with a custom-designed full-time Boston brace, between January 10, 2013 and October 23, 2018, as described in the original protocol and the approved ethical application. All patients were treated according to the SRS and SOSORT criteria [14] and identical inclusion and exclusion criteria were used in the 2 groups.

### Treatment

The Boston scoliosis night brace [15] is a custom-fabricated hyper-corrective nighttime brace. It is worn only while in bed (8 hours) and works by applying 3-dimensional counterforces stabilizing lateral shift and unbending forces on the curve. The Boston full-time brace [16] is a custom-fabricated orthosis. It corrects the scoliotic curve by applying 3-dimensional counterforces through pads on the convex side of the curve at the level of the apex and below. An orthotist was available for brace adjustment when needed. Introduction and adjustment of the brace were conducted in an outpatient or inpatient setting by the same orthotist at each site throughout the study period.

### Patients

Individuals were informed about the study if they met the following criteria: untreated idiopathic scoliosis, 9–17 years of age, estimated remaining growth in body height for at least 1 year, Cobb angle of 25°–40°, curve apex at thoracic vertebra 7 or caudal and for girls not more than 1 year after menarche. Exclusion criteria were non-idiopathic scoliosis, previous brace or surgical treatment for scoliosis, or inability to understand Swedish.

In both groups, transition to the other type of brace type was possible. Individuals in the nighttime brace group were offered transition to a full-time brace in the event of curve progression of > 6° seen on 2 consecutive radiographs compared with the radiograph at the time of inclusion [5]. Individuals in the full-time brace group were allowed transition to a nighttime brace if they were unwilling to continue treatment with the full-time brace.

Individuals in the nighttime brace group were, as part of the randomized controlled trial, radiographed twice yearly until skeletal maturity. Individuals in the full-time brace group were radiographed once yearly until skeletal maturity. All patients were followed at least until skeletal maturity unless surgery occurred before maturity. Information concerning any scoliosis surgeries was recorded until December 31, 2023 from the patient’s medical charts.

The study was reported according to the STROBE guidelines.

### Collected data

Patient demographics including age, sex, menarchal status, angle of trunk rotation (ATR) measured by a scoliometer, bodyweight, and height were collected at baseline. Radiographic analyses included: (i) most recent posteroanterior (PA) standing radiograph prior to brace start, (ii) in-brace supine or standing PA radiograph after treatment start, (iii) 24 hours out-of-brace PA standing radiograph at the last follow-up prior to brace termination or the last PA standing radiograph post brace termination if available, and (iv) sagittal parameters such as thoracic kyphosis T5–T12 and lumbar lordosis L1–S1. All radiographic analyses were performed by 2 independent experienced physicians, blinded to treatment intervention, after study termination. All measurements were conducted through radiographic images in Digital Imaging and Communications in Medicine (DICOM) image format using the PACS clinical imaging tool (Sectra PACS, version 23.1, Linköping, Sweden) and the Surgimap software (Surgimap Spine Software, version 2.3.2.1, Nemaris Inc, New York, USA).

### Outcome

The type of curvature (thoracic, thoracolumbar, lumbar) was recorded, and the magnitude of the main curve was measured according to the Cobb method. In-brace correction was calculated according to the formula: (Cobb angle on erect radiograph – Cobb angle on in-brace radiograph)/Cobb angle on erect radiograph) x 100%. Surgery was offered if curve sizes were 45° or larger.

### Statistics

Descriptive data are presented as mean (range or SD), number (%) or median with interquartile range (IQR). Visual estimation of quantile–quantile plots (Q–Q plots) was used to assess data distribution. Continuous variables were compared using unpaired, two-tailed t-tests. Categorical variables were compared using the Pearson chi-square test. Mann–Whitney U tests were used for non-normally distributed continuous data.

Binary logistic regression was used to assess the risk of major curve progression to ≥ 45° at the last available radiographic follow-up. Variables entered in the model were decided a priori and included: age, sex, curve size at the beginning of brace treatment, in-brace correction, menarchal status (for girls), and Risser grade. Statistical significance was set at P < 0.05. IBM SPSS statistical software version 28 was used for statistical analyses (IBM SPSS Statistics for Windows, Version 28.0; IBM Corp, Armonk, NY, USA).

### Ethics, fundings, use of AI, and disclosure

Approval was obtained by the Regional Ethical Board in Stockholm (Dnr 2012/172-31/4 and 2015/1007-32). Grants were received from the Swedish Research Council and the Stockholm County council (ALF). The funding sources had no role in the study design, analysis, or interpretation of data, in the manuscript writing, or in the decision to submit the paper for publication. No benefits in any form have been received or will be received from a commercial party related directly or indirectly to the subject of this article. AI was not used in the preparation of this manuscript. Complete disclosure of interest forms according to ICMJE are available on the article page, doi: 10.2340/17453674.2025.43706

## Results

The flowchart of the study is shown in the Figure. Of the screened individuals, 202 were found to be eligible. 135 consented to randomization and 67 declined. Of those randomized, 45 were allocated to a nighttime brace. In the 67 who declined randomization, another 22 declined a full-time brace. This left 44 patients preferring full-time bracing.

**Figure:**
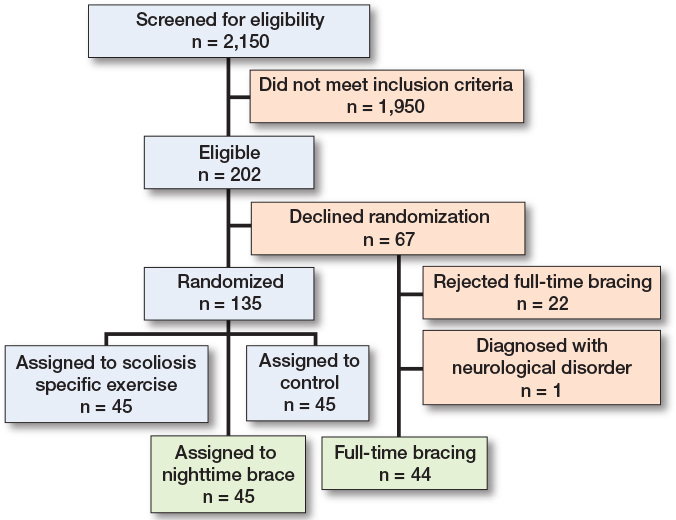
Flowchart of the study.

At baseline, the median age for the entire cohort was 12.8 (IQR 1.8) years (Table 1). Age, sex, BMI, menarche for girls, and Risser grade did not differ significantly between the 2 groups (all P ≥ 0.1), while angle of trunk rotation (ATR) was statistically significantly higher in the nighttime brace group (Table 1). There were no statistically significant differences between the 2 brace groups with regards to location and magnitude of the major curve, thoracic kyphosis, and length of brace treatment (all P ≥ 0.06). In-brace correction of the major curve was 48% in the nighttime and 51% in the full-time brace groups (P = 0.06). The nighttime brace group demonstrated a statistically significant greater lumbar lordosis than the full-time brace group (P = 0.02) (Table 2).

**Table 1:** Baseline characteristics for the entire cohort and the 2 groups. Data shown as median (IQR), or number (%). n = number of patients with available data

Item	Overall n = 89	Night time brace n = 45	Full-time brace n = 44	P value
Age	12.8 (1.8)	12.8 (2.0)	13.0 (1.0)	0.4 ^**c**^
Females, n (%)	76 (85)	39 (44)	37 (41)	0.7 ^**d**^
Height, cm	157 (13)	157 (11)	159 (16)	0.1 ^**c**^
Weight, kg	45 (13)	45 (13)	45 (14)	0.8 ^**c**^
Body mass index	18 (3)	18 (3)	18 (3)	0.9 ^**c**^
Premenarcheal, n (%) ^**a**^	44 (58)	22 (29)	22 (29)	0.6 ^**d**^
Angle of trunk rotation, °	10 (4)	11 (4)	10 (5)	0.03 ^**c**^
Risser grade, n (%) ^**b**^				0.5 ^**d**^
0	31 (44)	21 (50)	10 (36)	
I	9 (13)	5 (12)	4 (14)	
II	9 (13)	5 (12)	4 (14)	
III	19 (27)	9 (21)	10 (36)	
IV	2 (2.9)	2 (5.0)	0	
V	0			

a Missing or non-applicable for male patients.

b Risser grade is a staging system of bone maturity based on the ossification of the iliac apophysis. It ranges from 0 to 5; higher grades indicate greater maturity. Risser grading was not necessary for treatment initiation and was therefore missing in some individuals.

c Mann–Whitney U test.

d Pearson chi-square test.

**Table 2:** Radiographic comparison between the nighttime and the full-time brace group. Data shown as median (IQR), or number (%). n = number of patients with available data

Item	Overall n = 89	Nighttime brace n = 45	Full-time brace n = 45	P value
Pre brace				
Cobb angle of the major				
curve, °		32 (6)	33 (5)	0.8 ^**c**^
Location of the major curve, n (%)				0.3 ^**b**^
Thoracic	56 (63)	31 (69)	25 (57)	
Thoracolumbar	18 (20)	9 (20)	9 (20)	
Lumbar	15 (17)	5 (11)	10 (23)	
Thoracic kyphosis, °		15 (11)	17 (9)	0.2 ^**c**^
Lumbar lordosis, °		–50 (15)	–48 (9)	0.02 ^**c**^
In brace correction (%)		48 (26)	51 (21)	0.06 ^**c**^
Post brace				
Months in the brace		24 (15)	27 (10)	0.4 ^**a**^
Months post brace		21 (8)	18 (9)	0.3 ^**c**^
Cobb angle of the major curve				
at last available radiograph, °		39 (11)	38 (11)	0.7 ^**c**^

a Mann–Whitney U test.

b Pearson chi-square test.

c 2 sample t-test.

During the brace treatment period, 10 individuals transitioned to a full-time brace in the nighttime brace group, and 7 individuals transitioned to a nighttime brace in the full-time brace group (P = 0.5).

### Outcome

The median radiographic follow-up time after brace start and until the last available radiograph did not differ significantly between the nighttime and the full-time brace group (33 [IQR 24] vs 33 [IQR 27] months; P = 0.9). At that endpoint, there were no significant differences between the 2 brace groups with regards to the magnitude of the major curve (P = 0.7) (Table 2).

16 patients in the nighttime brace group and 14 in the full-time brace group demonstrated curve progression to ≥ 45° at the last available radiographic follow-up (P = 0.7). In the binary logistic regression, female sex (odds ratio [OR] 6.5, 95% confidence interval [CI] 1.1–37.4), lower Risser grade (OR 1.6, CI 1.01–2.7), and larger curve size at the beginning of brace treatment (OR 1.4, CI 1.2–1.5) had higher odds for curve progression to ≥ 45°. The model explained 45% (Nagelkerke R2) of the variance and correctly classified 79% of cases.

The mean total follow-up time was 94 (IQR 20) months with a range of 62–131 months. During this period, 11 patients in the nighttime brace group and 6 patients in the full-time brace group underwent scoliosis surgery (OR 2.0, CI 0.7– 6.1). The mean Cobb angle of the major curve at the time of surgery was 51° (IQR 6) in the nighttime brace group and 59° (IQR 3) in the full-time brace group (P = 0.002). The most common curve type was thoracic (n = 12) followed by thoracolumbar (n = 4). The mean time from brace start to surgery did not differ significantly between the 2 groups: 33 (IQR 18) vs 31 (IQR 13) months; P = 0.2, respectively.

## Discussion

This is the first prospective multicenter study exploring the effectiveness of nighttime brace vs full-time brace and presents several advantages. We aimed to investigate risk of curve progression and risk of surgery between nighttime bracing and full-time bracing for patients with moderate-grade adolescent idiopathic scoliosis. We showed similar effectiveness of nighttime bracing and full-time bracing in the prevention of curve progression and a tendency towards more surgical interventions in the night brace group.

The evidence on the effectiveness of nighttime braces is scarce and few studies have compared nighttime vs full-time bracing [8-10]. The results of these studies varied, mainly due to inconsistent inclusion criteria and definition of brace effectiveness. Recently, Ohrt-Nissen et al. [11] reported on the effectiveness of a nighttime brace and full-time brace and reported similar effectiveness for the 2 treatment options. In line with Ohrt-Nissen et al. are the results of a recently published metanalysis and a systematic review based on SRS criteria; both studies reported similar effectiveness of nighttime and full-time braces [17,18].

Similar to previous studies [11,19,20] was the progression rate to surgical magnitude seen in our study; 34% of the patients in this cohort had curve progression equal to or beyond the threshold of 45°, where surgery can be considered as an option. Although patients in the full-time brace group had larger curves at the time of surgery, the nighttime brace group showed a trend toward a higher rate of surgical intervention. Nevertheless, the comparable rates of progression to the surgical threshold (≥ 45°) in both groups suggest that factors beyond the effectiveness of the brace—such as clinical judgment, patient preference, or curve characteristics—may have influenced the decision to proceed with surgery. The results of our study are in line with previously published studies that showed similar effectiveness between nighttime and full-time bracing [11,17,21] and support the use of a night brace as an alternative to a full-time brace. In cases where problems with treatment compliance in a full-time brace can be expected [1] the nighttime brace may be an alternative suggestion.

Despite the use of a hypercorrective design, the in-brace Cobb angle correction in the nighttime group was modest and similar to that of the full-time brace group (48% and 51%, respectively). In-brace correction for full-time braces has been reported as being from 30% to 50% [22-24], in line with our results. In contrast, in-brace correction in the nighttime braces has been reported in the literature as being from 61% to 96% [25,26]. Other investigators suggested a minimum 70% in-brace correction to be considered in nighttime braced patients [27]; this is quite a bit higher than the in-brace correction seen in this study. The reason for this is unclear, as introduction and approval of in-brace correction was conducted by the same senior orthotist who oversaw all sites throughout the study period. It may reflect limitations in brace application, patient-specific variability, or suboptimal fit in some cases. Nevertheless, our results showed similar effectiveness of a nighttime brace as compared with a full-time brace, despite a lower than suggested in-brace correction. Additionally, in our regression model, in-brace correction did not significantly affect curve progression to ≥ 45°.

In the current study, sex, skeletal age and curve size at the beginning of brace treatment were found to be predictors of curve progression in patients with adolescent idiopathic scoliosis. Several studies have reported on factors associated with curve progression in patients with idiopathic scoliosis. Similar results to the current study have been presented by other investigators [11,28-30], all highlighting initial curve size and skeletal immaturity as factors associated with curve progression.

### Limitations

First, there was no measure of brace compliance during the treatment period. We cannot exclude that unmeasured confounding may have influenced the results of this study as the comparisons are not based on randomized groups. However, there were no significant differences in baseline scores in variables of importance, suggesting that the 2 groups were fairly similar and comparable. Nevertheless, we believe that this is a real-world study, reflecting the actual effect of brace treatment. Thus, all patients with intent to treat were included in the analysis; in fact, the SRS and SOSORT recommend including all patients in the intention to treat analysis, regardless of the level of compliance.

Second, patients in the nighttime brace group, as part of a randomized controlled trial, adhered to a different follow-up protocol and were radiographed twice a year while individuals in the full-time brace group were radiographed once a year. Similarly, no patient-reported outcomes were available in the full-time brace group and therefore it is unknown whether differences in treatment satisfaction existed between the groups.

Third, in the RCT protocol individuals in the nighttime brace group were transitioned to a full-time brace if progression occurred, while those in the full-time brace group could choose to transition to a nighttime brace. The rates for these transitions were similar in the 2 groups and may reflect the real-world situation but obviously make interpretation of the results difficult.

Fourth, although brace adjustments to optimize fit and correction were performed as part of routine clinical care, the number and type of adaptations were not recorded. This represents a limitation in interpreting the potential influence of brace optimization on treatment outcomes.

Fifth, derotation at the apex of the curve, although expected with hypercorrective brace designs, was not systematically assessed in this study. Future studies should incorporate standardized axial rotation measures to fully characterize in-brace correction.

Sixth, the study was not powered to detect differences between curve types in either the primary outcome or this secondary analysis and does not allow for conclusions as to whether different brace designs may have varying effects on different curve types. However, both groups were sampled from a consecutive population sampling methodology, which suggests generalizability to the Swedish national population and similar healthcare systems and populations abroad. Therefore, the sampling does not undermine the ability to explore potential differences in the effects associated with the interventions on the samples. Moreover, the sample size may be too small to identify differences in treatment effectiveness. Lastly, we cannot exclude that nighttime bracing would have been more effective if we had attained higher grades of in-brace correction, equivalent to previous studies [25-27].

### Conclusion

Nighttime bracing including a possibility to transition to a full-time brace in case of progression demonstrated comparable effectiveness in preventing curve progression, but a tendency to a higher risk of surgical treatment.

## Notes

Conceptualization: PG, AA, HM; methodology: PG, ED, AA, HM, AC; formal analysis: AC, ED; investigation: AC, ED, KJ, AO, AAA, HM, AA, PG; resources: PG and AA; data curation: AC, ED, KJ, AO, AAA, HM, AA, and PG; writing original draft preparation: AC; writing review and editing: AC, ED, KJ, AO, AAA, HM, AA, and PG; visualization: AC; supervision: AA, HM, PG; project administration: AA and PG; funding acquisition: PG and AA. All authors have read and agreed to the published version of the manuscript.

The authors would like to thank Ingrid Ekenman, MD, PhD (retired); Tomas Reigo, MD, PhD (retired); Peter Endler, MD, PhD (Stockholm Center for Spine Surgery); Sofia Berto, RPT (Kry Healthcare Center); Suzanne Torén, RPT (Karolinska University Hospital); Panayiotis Savvides, MD (Karolinska University Hospital); Ylva Bodén, Linköping University Hospital; and Björn Dahlman, MD (Västmanlands Hospital in Västerås), who recruited patients. Luigi Belcastro, RNP (Karolinska University Hospital), and Maria Wikzen, RNP (Karolinska University Hospital), were research coordinators. There was no financial compensation for these contributions.

Handling co-editor: Ilkka Helenius

*Acta* thanks James O Sanders, Ane Simony and other anonymous reviewers for help with peer review of this manuscript.
